# The First Evaluation of a Geroscience Biomarker Index (TAME-BI) in a Trial of Caloric Restriction and Exercise

**DOI:** 10.1093/geroni/igab046.299

**Published:** 2021-12-17

**Authors:** Jamie Justice, Mark Espeland, Denise Houston, Stephen Kritchevsky, Barbara Nicklas, Nicholas Pajewski, Dalane Kitzman

**Affiliations:** 1 Wake Forest School of Medicine, Wake Forest School of Medicine, North Carolina, United States; 2 Wake Forest School of Medicine, Winston-Salem, North Carolina, United States; 3 Wake Forest School of Medicine, Winston Salem, North Carolina, United States

## Abstract

We leveraged the WF OAIC biorepository to measure a consensus-derived panel of blood-based biomarkers of aging and constructed a geroscience-guided biomarker index (TAME-BI), testing it for the first time in a clinical trial. We measured IL-6, TNF-α-receptor-I, growth differentiating factor-15, cystatin C, and N-terminal pro-B-type natriuretic peptide in a 20-week randomized trial of caloric restriction (CR), aerobic exercise (EX), CR+EX, or attention-control in 88 patients (67±5years) with heart failure with preserved ejection fraction (HFpEF). We calculated TAME-BI (analyte levels ranked, binned by quintile, and summed) and found a time×treatment interaction for improved TAME-BI with intervention (p≤0.05) and detected associations between change in TAME-BI and change in six-minute walk distance (r= -0.24), usual walk speed (r= -0.23), and left ventricular relative wall thickness (r= 0.31). In sum, CR+EX intervention improved TAME-BI and changes in TAME-BI were associated with changes in key functional measures in older HFpEF patients.

